# Intersphincteric Resection and Coloanal Anastomosis in Treatment of Distal Rectal Cancer

**DOI:** 10.1155/2012/581258

**Published:** 2012-05-29

**Authors:** Gokhan Cipe, Mahmut Muslumanoglu, Erkan Yardimci, Naim Memmi, Erhan Aysan

**Affiliations:** Department of General Surgery, Faculty of Medicine, Bezmialem Vakif University, Adnan Menderes Bulvari, Istanbul 34090, Turkey

## Abstract

In the treatment of distal rectal cancer, abdominoperineal resection is traditionally performed. However, the recognition of shorter safe distal resection line, intersphincteric resection technique has given a chance of sphincter-saving surgery for patients with distal rectal cancer during last two decades and still is being performed as an alternative choice of abdominoperineal resection. The first aim of this study is to assess the morbidity, mortality, oncological, and functional outcomes of intersphincteric resection. The second aim is to compare outcomes of patients who underwent intersphincteric resection with the outcomes of patients who underwent abdominoperineal resection.

## 1. Introduction

Colorectal cancer is the third most common cancer and the fourth leading cause of cancer death worldwide. It is also the second most common cancer in women and the third most common in men within European countries [1]. Although colon cancer and 2/3 proximal rectal cancer are treated more easily, treatment of distal rectal cancer involves challenges even colorectal surgeons. Abdominoperineal resection (APR) has been the usual treatment option for distal rectal cancer since Miles reported this technique in the 1920 [2]. However, APR inevitably includes permanent colostomy. Total mesorectal excision technique was described by Heald and Ryall and this is the gold standard management of middle and distal thirds of rectal cancer now. This technique both reduced the recurrence rate and increased the survival of the rectal cancer [3]. In addition, further studies suggested that distal intramural spread of rectal cancer rarely extends more than 1 cm beyond the distal margin of the tumor [4, 5]. Therefore, along with advances in preoperative chemoradiation therapy, a 1 cm distal margin has increased the incidence of successful sphincter-saving surgery [6]. Schiessel et al. first reported the intersphincteric resection (ISR) technique which has been used to increase sphincter preservation by achieving necessary distal margin for patients with distal rectal cancers [7]. Today, ISR and coloanal anastomosis are commonly preferred surgical treatment options of distal rectal cancer. The aim of this paper is to evaluate the mortality and morbidity, oncologic and functional outcomes after ISR for distal rectal cancer.

## 2. Materials and Methods

A literature search of Medline, Embase, Ovid, and Cochrane database was performed to identify relevant articles in the English language associated with ISR for rectal cancer for the years 1960 to 2012.

## 3. Surgical Technique

MRI and EUS are commonly used preoperative staging rectal cancer. In addition these two modalities, in evaluating whether a distal rectal cancer is eligible for ISR surgeons, use rigid proctoscopy and digital assessment of the level of the tumor in relation to the anal sphincter. Neoadjuvant treatment is performed T3, T4, and N positive rectal cancer for down staging and increase of possibility of sphincter-saving surgery. Common practice is performed to surgery within 6 weeks after neoadjuvant therapy [8]. 

 The indication for ISR is any type of distal cancer extending or involving the anal ring. The internal anal sphincter involvement is also included. The tumors invading external anal sphincter or levator ani muscle and T4 cancers did not respond to neoadjuvant therapy, involving the prostate or vagina, preoperative poor sphincter functions are contraindications of ISR. The most common indication for ISR is cancer within 1 cm of the anorectal ring. ISR and coloanal anastomosis are performed as both abdominal and perineal approach. Abdominal part of the operation is performed either as open or laparoscopic technique [9–11]. 

**Figure 1:**
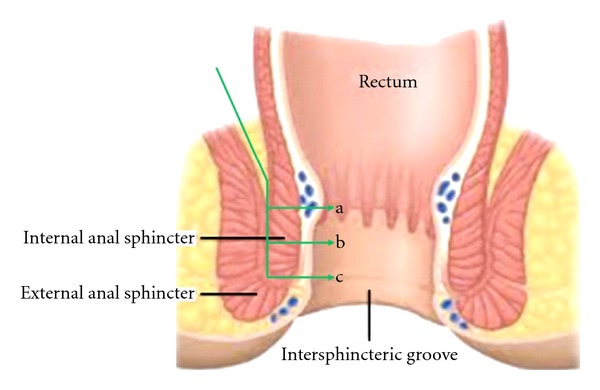
Type of ISR according to amount of excision of the internal anal sphincter. a: partial ISR, b: subtotal ISR, and c: total ISR.

The first step of abdominal part is high ligation of inferior mesenteric artery and left colonic mobilization including takedown of splenic flexure almost all patients. Second step is total mesorectal excision, with sharp dissection along an embryologic plane between the mesorectal fascia and the fascia of the pelvic sidewall and preserving hypogastric plexus nerves according to the method described by Heald [12]. The dissection is performed as distal as possible and the puborectal muscle surrounding lateral and posterior wall of the rectum is exposed at the pelvic floor to facilitating the perineal dissection. The first step of the perineal part of the operation is good exposition of the anal canal via self-retaining retractor (Lone Star Retractor; Lone Star Medical Products Inc., Houston, TX, USA). After injecting 1 mg diluted epinephrine in 20 mL of saline solution which minimized bleeding and facilitating intersphincteric dissection, the mucosa and internal sphincter are circumferentially incised at least 1 cm distance from the distal edge of the tumor. The anal orifice is then closed transanally with pursestring sutures to prevent tumor cell dissemination during the perineal approach. There are 3 types of ISR, called total, subtotal, and partial. When the tumor spread beyond the dentate line, total ISR should be done. The internal sphincter is completely removed, and the distal margin of resection is at the intersphincteric groove. If the distal edge of the tumor is more than 2 cm far from dentate line, subtotal ISR is performed instead of total ISR. The distal resection margin of subtotal ISR is between dentate line and the intersphincteric groove. If the surgeon has a enough distal surgical margin, the distal line of the resection can be on or above the dentate line. This is called parial ISR. The descriptions of 3 type of ISR are shown in Figure 1. Dissection continues through intersphincteric plane to connect with dissection from abdomen.

After the rectum is totally separated from prostate or vagina, the specimen is removed per anally. Frozen-section histopathology should confirm the lack of tumor cells in the distal margin. Colonic J pouch, transverse coloplasty, or straight coloanal hand-sewn anastomosis can be performed according to surgeons preference. However, the latter associated with high incidence of tenesmus, urgency, and incontinence [13]. Pelvic drain is placed, and defunctioning stoma is created in most of patients.

## 4. Results

### 4.1. Morbidity and Mortality

ISR and coloanal anastomosis associate with complications and mortality like any other colorectal operations. Mortality rate of within postoperative 30 days was reported between 0 and 6 percent of patients in the different studies and is shown in Table 1. The common causes of death both surgery related factor (e.g., anastomotic leak) and consequence of comorbid medical conditions (myocardial infarction, pulmonary embolus) have been reported in the recently published meta-analysis [14]. 

The common complications of ISR are anastomotic leakage, stricture, fistula, pelvic sepsis, bleeding, bowel obstruction, and wound infections, which have been reported in different studies and are shown in Table 1. Anastomotic leaks are inevitable complications that have been previously reported to affect 2.6% and 24% of patients undergoing colorectal surgery [39, 40]. Likewise, the most serious complication of ISR and coloanal anastomosis is anastomotic leakage. Anastomotic leakage was defined by the presence of a pelvic abscess and was confirmed by a computed tomography scan or clinical peritonitis. Once the anastomotic leakage is diagnosed, prompt management has a vital significance. Although diverting loop ileostomy is a common surgical choice to secure an anastomosis or to divert feces from a distal affected intestinal segment, it has become clear that an anastomotic leak cannot be prevented by a proximal diversion, but septic symptoms can be reduced [41]. Anastomotic leakage has been reported 0.9–13% of ISR surgery in the different studies. The rate of pelvic sepsis is reported up to 5 percent, majority of these originate from an anastomotic leak [25]. Intraoperative blood transfusion and pulmonary disease were found to be independent risk factors for anastomotic leakage in the recent study [28].

Anastomotic leakage is managed by diverting ileostomy (if not perform initial operation) or percutaneous drainage. If the cause of anastomotic leakage is ischemic distal segment, pouch excision and reanastomosis or stoma creation with APR may be required.

Intestinal obstruction was defined by a combination of the following findings: abdominal distention, abdominal pain, vomiting, and the presence of air-fluid levels on a plain abdominal radiograph during the postoperative period. Postoperative intestinal obstruction is presented between 0–16% according to various studies, and most of the patients manage conservatively [21, 32]. Failure of the conservative management requires further surgery in a few patients.

Wound infection is the most common minor complication of the ISR surgery. Wound infection is defined by the presence of purulent discharge, erythema, and induration of the wound. Wound infection has been reported up to 9 percent (Table 1). All of the wound infections were treated successfully by open wound care.

### 4.2. Oncologic Outcomes

#### 4.2.1. Locoregional Recurrence

The local recurrence rates of different studies regarding intersphincteric resection are summarized in Table 2. The rates of isolated local recurrence reported are between 2% and 31% in these studies.

Various studies have shown that intersphincteric resection does not increase local recurrence rates [31].

Recurrence rate of the distal rectal cancer was radically reduced by total mesorectal excision technique which was first reported by Heald et al. Today the most of local recurrence is considered as being incomplete of surgical excision. However, involvement of circumferential resection margin is associated with high recurrence rate even if TME is properly performed [36]. In addition, some authors argue that involvement of lateral pelvic lymph node is responsible up to 22% of locoregional recurrence [47].

Another important point of local recurrence is tumor shedding. Cancer cells have been found on the peritumoral tissue and doughnuts after stapling anastomosis [48]. Because handling of the rectum during surgery causes increased number of cancer cells shed, no touch technique can be beneficial [49].

#### 4.2.2. Survival

Range of the 5-year overall survival rate of intersphincteric resection was 62%–97%, and disease-free survival was 66%–87% in the different studies. (Table 2). Recently published study has reported that 5-year overall survival for patients after ISR was 80%, and disease-free survival was 69.1%. These results considered better than 5-year overall survival of APR but not 5-year disease-free survival [31].

Kuo et al. analyzed the comparison between low anterior resection and stapled colorectal anastomosis, radical proctectomy with ISR and APR. The authors found significantly differences in overall survival among three groups and APR had statistically shorter survival than others [46]. All these results suggest that intersphincteric resection is a safe procedure in terms of oncologic outcomes.

### 4.3. Functional Outcomes

Preservation of the sympathetic and parasympathetic nerves is one of the most important part of the TME in the rectal cancer surgery. There are four zone nerve damages that can occur. First, the root of the inferior mesenteric artery (damage of sympathetic hypogastric nerve); second, posterior rectal plane (damage of sympathetic hypogastric nerve); third, lateral rectal plane (sympathetic and parasympathetic nerves); fourth, anterior rectal dissection (cavernous nerve). Damage of these nerves causes urinary dysfunction or impotence in most of patients [50].

Functional results of different studies are shown in Table 3. Jorge and Wexner incontinence score, the Kirwan classification system, and other institutional questionnaires are usually used to evaluate patients' functional results. Postoperative functional outcomes seem to be acceptable. Incontinence was a record of the number of bowel movements in 24 hours almost in all studies. The bowel movement rates from 2.2 to 3.7 per 24 hours and fecal soiling rate from 11% to 59% are reported. Rullier et al. show that if more than half of the internal sphincter is resected, incontinence is worse but remains normal in 50% patients [8]. Denost et al. investigated risk factors fecal incontinence after ISR in 101 rectal cancer patients and they found that the only independent predictors of incontinence were distance of the tumor lower than 1 cm from the anal ring (*P* = 0.004) and anastomoses lower than 2 cm above the anal verge (*P* = 0.037) [51]. It should be considered that functional outcomes may be improved by use of J pouch or coloplasty [52]. Before surgery, the patient must be informed about possible functional outcomes of intersphincteric resection.

### 4.4. ISR versus APR

Although there are numerous studies comparing sphincter-saving surgery and APR [44, 53], few studies were found regarding comparison of ISR and APR due to heterogeneity of the sphincter-saving surgery groups. These studies are summarized in Table 4.

The study of Weiser et al. concluded that patients undergoing APR were elder (*P* = 0.0006) and have more poorly differentiated tumors (*P* = 0.03). Although there was no statistical significant difference in the pretreatment endorectal ultrasound stage, APR was associated with poorer outcome in this study. Saito et al. reported that though a significant difference in overall survival was observed, there was no significant difference in disease-free survival between ISR and APR groups. The authors concluded that ISR appears to be oncologically acceptable and can reduce the number of APRs [31]. 

## 5. Discussion

Multimodality treatment has brought advances in treatment of locally advanced rectal cancer during the last two decades. The Swedish Rectal Cancer Trial, assessing preoperative short course radiotherapy, found a benefit in overall survival compared to surgery alone [54]. In addition to this benefit, preoperative radiotherapy provides downsizing and downstaging which increase possibility of sphincter-saving surgery in patients with distal rectal cancer. Preoperative radiotherapy or chemoradiotherapy should be recommended for T3-4 or N1 rectal cancers [8].

Distal rectal cancer is considered surgical challenge even by colorectal surgeons. The ISR technique is a valuable sphincter-saving surgical treatment in patients with distal rectal cancer. Patients selection for ISR is based upon careful preoperative staging. The level of the transection of the internal sphincter should be decided before surgery. Detection of preoperative external sphincter invasion or fecal incontinence is all contraindication of ISR. In addition, some authors argue that ISR is contraindicated to poorly differentiated or mucinous cancer [7, 55].

Recently published systematic review reported that the overall mortality associated with ISR is 0.8%. The overall morbidity rate reported is 25.8%. Anastomotic leak was experienced after a mean of 9.1% and the pelvic sepsis rate was of 2.4% [14].

Postoperative overall morbidity rate varies between series from 8% to 64%. Anastomotic leak rates are reported of 0.9–48%. (Table 1) this difference arises from some studies that include the asymptomatic leakage which is radiologically detected. Akasu et al. reviewed 120 patients who underwent ISR and reported risk factors for anastomotic leakage following ISR. This study suggests that intraoperative blood transfusion, pulmonary disease, and colonic J-pouch are independent risk factors for leakage following ISR [28].

One of the main targets of surgical treatment of rectal cancer is as possible as long disease-free survival. Therefore, the most important question to answer is ISR technique carries an increased risk local recurrence or decline survival. In the various studies, range of the 5-year overall survival rate of intersphincteric resection was reported from 79% to 97%, and disease-free survival was reported from 69% to 87%. (Table 2).

Tilney and Tekkis reported review, including 21 studies accumulating a total of 612 patients who underwent ISR for distal rectal cancer. The mean 5-year survival following ISR was reported in 81.5%. Locoregional recurrence rate was available from all of the studies evaluated for oncologic outcomes, with 51 of 538 patients (9.5%) experienced local recurrence [56].

Akasu et al. investigated risk factors for local and distant recurrence in 122 patients. Local recurrence rate found 6.7% and distant recurrence rate was found 13%. Positive resection margins, dedifferentiation of tumor, and elevated preoperative levels of CA19-9 (>37 U/mL) were reported risk factors of local recurrence. Pathological N1, N2 tumor, poor differentiation, and the tumor close to anal canal less than 2.5 cm were reported risk factor for distant recurrence [27].

The current systematic review and meta-analysis which included 14 studies reported that the mean distal margin free from tumor was 17.1 mm, CRM-negative margins were achieved in 96% of patients, RO and the overall local recurrence rate were 6.7% (range: 0–23%). The 5-year overall and disease-free survival rate was 86.3% and 78.6%, respectively [14]. The authors conclude that available datas with potential for selection bias, oncological outcomes after ISR are affected negatively.

There are limited studies in the literature about functional outcomes after ISR (Table 3). Jorge and Wexner incontinence score and the Kirwan classification system were generally used for evaluating patient' functional outcome after ISR. Although neoadjuvant chemoradiotherapy has a beneficial effect downsizing and downstaging in patients undergoing ISR, it probably has a negative effect on functional results. Canda et al. showed that neoadjuvant chemoradiotherapy was associated with significantly lower maximal squeeze pressures and worsening of Wexner scores who had received neoadjuvant chemoradiotherapy [57]. This data support that counseling patients about expected functional outcomes is important.

Current metaanalysis of 8 studies demonstrated that the mean number of bowel motions in a 24 h period was 2.7, 51.2% patients experienced “perfect incontinence”, 29.1% patients experienced fecal soiling. Incontinence to flatus is reported by 23.8% in this study [14]. However, Bretagnol et al. reported that the Wexner score and the Fecal Incontinence Severity Index (FISI) were significantly improved following colonic j-pouch reconstruction compared with straight coloanal anastomosis [58].

Quality of life after ISR has been rarely reported. Bretagnol et al. demonstrated that fecal incontinence-related QoL scores were poorer than LAR after ISR. However, SF 36 scores were similar [58]. Barisic et al. showed that fecal incontinence improved by the time and 11.1% patients had fecal incontinence after 1-year ISR. Moreover, most of patients had acceptable QoL scores according to all functional and symptom components of the European Organization for Research and Treatment of Cancer QoL-C30 questionnaire [59].

Kuo et al. reported functional outcomes of ISR in 162 patients; 38% had stool fragmentation, 23.8% had nocturnal defecation they reported and one-third needed antidiarrheal medications. However, 90.8% of patients was satisfied with functional results of ISR [46].

A few studies were found in the literature regarding comparison of ISR and APR (Table 4). Almost all studies reported low local recurrence rate and better survival for ISR technique. All of these studies have retrospective characters, and there could be bias about selection of the patients. However, only one study reported significant difference between ISR and APR by stage of rectal cancer [46]. Among these studies, 5-year survival was compared between ISR and APR by only one study regarding the stage of tumor. This study reported that according the Dukes' classification, 5-year survival rates for stages A, B, and C are 84%, 58%, and 27%, respectively, for ISR patients and 83.5%, 53%, and 37%, respectively, for APR patients [15]. Saito et al. published the well-designed-study in this area. Although there were no difference in patients' age (*P* = 0.662), gender (*P* = 0.187), and preoperative T (*P* = 0.798) and N (*P* = 0.521) stage, significant difference in overall survival was observed (*P* = 0.033) but no significant difference in disease-free survival between two groups (*P* = 0.714). There is one weak point in this study that the most of the APR was performed between 1995 and 2002. Only 11 patients underwent APR between 2000 and 2006. The authors conclude that acceptable oncologic outcomes were gained with ISR, and the use of ISR can reduce the number of APRs in patients with distal rectal cancer [31].

## 6. Conclusion

The ISR technique provides an opportunity to perform sphincter-saving surgery in treatment of distal rectal cancer. The favorable tumor is early stage, well differentiated or has a good regression after neoadjuvant therapy. This technique performs with acceptable functional outcomes. Moreover, if the adequate distal margin is provided, the local recurrence and survival rates after ISR may even be better than those of APR. The ISR technique should be considered as a safe procedure and a valuable alternative to APR in selected patients with distal rectal carcinomas.

## Figures and Tables

**Table 1:** Complications and mortality after ISR.

Reference	Anastomotic leak (%)	Anastomotic stricture (%)	Fistula (%)	Pelvic sepsis (%)	Wound complications (%)	Bleeding (%)	Bowel obstruction (%)	Rectal mucosal prolapse (%)	Mortality (%)
Braun et al. [15] (1992)	10	3	0	0	8	0	3	NR	6
Bannon et al. [16] (1995)	0.9	0.9	0.9	0.9	1.8	0.9	0.9	1.8	1.0
Köhler et al. [17] (2000)	48	10	19	0	6	3	10	NR	0
Kim et al. [18] (2001)	6.2	6.2	4.2	NR	NR	NR	8.3	NR	NR
Tiret et al. [19] (2003)	11	NR	NR	3.8	NR	3.8	NR	NR	0
Luna-pérez et al. [20] (2003)	9.4	6.25	6.25	9.3	6.25	NR	6.25	NR	NR
Rullier et al. [21] (2005)	11	0	2	3	0	7	0	NR	0
Schiessel et al. [22] (2005)	NR	NR	5.1	NR	NR	0.8	NR	NR	0.8
Hohenberger et al. [23] (2006)	NR	NR	NR	NR	NR	NR	NR	NR	3
Saito et al. [24] (2006)	10.1	0	1.3	4.4	0	1.3	0	1.3	0.4
Chamlou et al. [25] (2007)	9	0	1	5	1	2	0	NR	0
Dai et al. [26] (2008)	NR	8.7	8.7	NR	NR	NR	NR	NR	0
Akasu et al. [27] (2008)	13.0	NR	NR	NR	NR	NR	NR	NR	0.8
Akasu et al. [28] (2010)	13	0.8	NR	NR	6.6	NR	5	0.8	0.8
Han et al. [29] (2009)	3	0	0	0	5	0	0	NR	0
Krand et al. [30] (2009)	4	2	0	2	9	0	2	NR	0
Saito et al. [31] (2009)	NR	NR	NR	NR	NR	NR	NR	NR	0
Weiser et al. [32] (2009)	5	16	5	0	7	0	16	NR	0
Yamada et al. [33] (2009)	4.7	8.4	0	0	3.7	0	8.4	3.7	0
Han et al. [34] (2010)	1.6	2.5	0.6	NR	NR	NR	NR	NR	NR
Park et al. [35] (2011)	6.2	1.3	NR	NR	NR	NR	2.5	NR	1.3
Lim et al. [36] (2011)	1.8	6.3	0.9	2.7	NR	NR	4.5	1.8	0
Bennis et al. [37] (2012)	7	NR	NR	NR	NR	1.6	2.69	NR	0.4
Reshef et al. [38] (2012)	NR	NR	NR	2.9	4.5	NR	NR	NR	0.7

**Table 2:** Oncologic results of ISR.

Reference	Year	*N*	Median followup	Local recurrence	5-year survival (overall)	5-year survival (disease free)
Braun et al. [15]	1992	63	80	11	62	NR
Marks et al. [42]	1993	52	50	14	85	NR
Bannon et al. [16]	1995	109	40	11.0	87	NR
Mohiuddin et al. [43]	1998	48	48	15	82	NR
Köhler et al. [17]	2000	31	82	10	79	NR
Kim et al. [18]	2001	48	26	4.1	NR	NR
Tiret et al. [19]	2003	26	39	3.4	NR	NR
Nakagoe et al. [44]	2004	184	47.4	9.5	NR	73.6
Rullier et al. [21]	2005	92	40	2	81	70
Yoo et al. [45]	2005	29	57	31	86.2	65.7
Schiessel et al. [22]	2005	121	94	5.3	88	NR
Hohenberger et al. [23]	2006	65	70	23	NR	NR
Saito et al. [24]	2006	228	41	3.6	92	83
Chamlou et al. [25]	2007	90	56	7	82	75
Dai et al. [26]	2008	23	31.5	8.7	NR	NR
Akasu et al. [27]	2008	120	42	6.7	91	77
Krand et al. [30]	2009	47	68	2	85	82
Han et al. [29]	2009	40	43	5	97	86
Yamada et al. [33]	2009	107	41	2.5	92	87
Weiser et al. [32]	2009	44	47	0	96	83
Saito et al. [31]	2009	132	40	10.6	80	69
Han et al. [34]	2010	310	84	11.6	66	NR
Lim et al. [36]	2011	111	29.4	5.4	NR	NR
Kuo et al. [46]	2011	162	55	7.7	83	76
Reshef et al. [38]	2012	986	60	3	71	69

**Table 3:** Functional results of ISR.

Reference	Year	*n*	Anal manometry	Functional tool	Bowel movements per 24 hours	Complete incontinence (%)	Incontinence to flatus (%)	Faecal soiling (%)	Urgency (%)
Braun et al. [15]	1992	63	No	Mayo clinic classification	2.2 (1–3)	75	17	15	22
Köhler et al. [17]	2000	31	Yes	General questionnaire	3.3 (NR)	30	11	63	NR
Kim et al. [18]	2001	48	No	Kirwan classification	4.4 (3–6)	NR	NR	NR	NR
Tiret et al. [19]	2003	25	No	NR	2.5 (NR)	50	23	27	19
Schiessel et al. [22]	2005	121	Yes	Williams and johnston classification	2.2 (1–9)	86.3	NR	13.7	NR
Yoo et al. [45]	2005	17	No	Cleveland clinic incontinence score	5.0 (2–9)	NR	17.6	41.2	58.8
Saito et al. [24]	2006	228	No	Jorge and wexner incontinence score and kirwan score	NR	32.7	29.1	29.1	NR
Chamlou et al. [25]	2007	90	No	Jorge and wexner incontinence score	2.3 (NR)	41	25	59	19
Yamada et al. [33]	2009	107	No	Jorge and wexner incontinence score and Kirwan score	3.7 (2–6)	42.3	NR	27.9	NR
Han et al. [29]	2009	40	No	Kirwan classification	2.7 (NR)	43	29	29	31
Krand et al. [30]	2009	47	No	Kirwan classification	2.3 (2–5)	80	9	11	2
Kuo et al. [46]	2011	22	No	Wexner incontinence score	4.7 (NR)	NR	NR	NR	19

**Table 4:** Comparison ISR versus APR in terms of morbidity and oncologic outcomes.

References	No of patients			Overall morbidity			Local recurrence			Overall survival			Disease free survival			Median followup (months)
	ISR %	APR %	*P*	ISR %	APR %	*P*	ISR %	APR %	*P*	ISR %	APR %	*P*	ISR %	APR %	*P*	
Braun et al. [15] 1992	65	77	NR	30	NR	NR	11	17	NR	62	53	NR	NR	NR	NR	79
Kasper et al. [54] (1998)	85	81	NR	NR	NR	NR	8.7	17	NR	71	55	NR	NR	NR	NR	60
Saito et al. [31] 2009	132	70	0.18	30.3	28.6	0.30	10.6	15.7	0.29	80	61	0.03	69%	63%	0.714	48
Weiser et al. [32] 2009	44	63	NR	38.6	34.9	NR	0	9	NR	96	59	NR	83%	47%	NR	47
Kuo et al. [46] 2011	26	23	NR	NR	NR	NR	0	3.8	NR	83	46	0.006	76%	42%	0.029	55
